# Effects of removal of dietary polyunsaturated fatty acids on plasma extravasation and mechanical allodynia in a trigeminal neuropathic pain model

**DOI:** 10.1186/1744-8069-5-8

**Published:** 2009-02-25

**Authors:** Yasmina B Martin, Carlos Avendaño

**Affiliations:** 1 Department of Anatomy, Histology & Neuroscience, Autonoma University of Madrid, Medical School, 28029 Madrid, Spain

## Abstract

**Background:**

Neuropathic pain (NP) is partially mediated by neuroinflammatory mechanisms, and also modulates local neurogenic inflammation. Dietary lipids, in particular the total amount and relative proportions of polyunsaturated fatty acids (PUFAs) of the ω-3 and ω-6 families, have been reported to modify the threshold for thermal and mechanical allodynia in the partial sciatic nerve ligation model of NP in rats. The effects of dietary lipids on other popular NP models, such as the chronic constriction injury (CCI), have not yet been examined. It is also unknown whether dietary PUFAs exert any effect on the capsaicin (CAP)-induced neurogenic inflammation under control or NP conditions. In this study we investigated these interrelated phenomena in the trigeminal territory, which has been much less explored, and for which not all data derived from limb nerves can be directly applied.

**Results:**

We studied the effects of a CCI of the infraorbital nerve (IoN) on the development of mechanical allodynia and CAP-induced plasma extravasation in rats fed either a regular diet (RD), or a modified diet (MD) with much lower total content and ω-3:ω-6 ratio of PUFAs. In rats kept on MD, mechanical allodynia following CCI-IoN was more pronounced and developed earlier. Extravasation was substantially increased in naive rats fed MD, and displayed differential diet-depending changes one and four weeks after CCI-IoN. When compared with basal levels (in naive and/or sham cases), the net effect of CCI-IoN on ipsilateral extravasation was a reduction in the MD group, but an increase in the RD group, effectively neutralizing the original intergroup differences.

**Conclusion:**

In summary, PUFA intake reduces CAP-induced neurogenic plasma extravasation in the trigeminal territory, and their removal significantly alters the mechanical allodynia and the plasma extravasation that result from a unilateral CCI-IoN. It is likely that this "protective" effect of dietary lipids is temporary. Also, the presence of contralateral effects of CCI-IoN precludes using the contralateral side as control.

## Background

Chronic pain syndromes occur frequently in the trigeminal territory. Even disregarding headaches and dental pain, prevalence of orofacial pain reached 25–30% in a population-based study in the UK [1]. The mechanisms of neuropathic pain (NP) are multiple and still not fully understood [2,3], and this knowledge is particularly deficient when it comes to chronic pain in orofacial regions. To date, a variety of neuropathic models in rodent's spinal nerves have been developed, mostly based on limited or partial injury inflicted at different nerve levels [4]. In the trigeminal system, on the other hand, only the chronic constriction injury of the infraorbital nerve (CCI-IoN) has gained wide acceptance as a rodent model for the study of trigeminal neuralgia [5,6]. Nociceptive behavior following CCI-IoN is characterized by mechanical allodynia and thermal hyperalgesia preceded by a transient phase of lower responsiveness to mechanical and thermal stimuli [5]. Except for a shorter onset latency of the positive symptoms, the CCI of the sciatic nerve leads to a similar outcome [7]. In addition, sciatic CCI results in a marked decrease of nerve stimulation-, substance P- or capsaicin (CAP)-induced local plasma extravasation in its cutaneous territory [8-11]. Whether this hallmark of neurogenic inflammation is likewise altered after CCI-IoN is still unknown.

The response to noxious stimulation is known to be influenced by diet, but this has been mostly proven for acute responses [12-15]. A series of more recent studies, however, showed that consumption of a soy-rich diet prevents the development of tactile allodynia and thermal hyperalgesia after a partial sciatic nerve ligation injury [16,17]. More specifically, dietary corn or soy fats suppressed tactile allodynia and heat hyperalgesia, whereas soy and casein proteins decreased heat hyperalgesia but had no effect on tactile allodynia [18]. Dietary lipids and, in particular, the polyunsaturated fatty acids (PUFAs) in the diet are known to affect many physiological processes, such as inflammation, hemostasis, vascular tone, and immune reactions, as well as a number of pathological conditions in which chronic inflammation and/or immune reactions are involved [19,20]. Not surprisingly, the value of food rich in PUFAs or of PUFAs supplements to relieve the pain that accompanies those conditions has been under sustained scientific scrutiny along the last decades [21,22].

No data are available concerning the neurogenic inflammation and the effects of dietary lipids for the trigeminal territory. And it must be noted that the results derived from experimental studies on the spinal systems cannot be uncritically extrapolated to the trigeminal system, because not all properties of the spinal ganglia and cord are directly applicable to the trigeminal ganglion and brain stem nuclei [23-27]. Hence the importance of characterizing the specific features of NP in a trigeminal model in order to better understand the mechanisms of orofacial pain syndromes. Within this context, the purpose of this study was to investigate, first, the effects of the CCI-IoN on neurogenic inflammation, as measured by the degree of Evans Blue (EB) extravasation after topical application of capsaicin on the vibrissal pad, and second, to examine the influence of altering the PUFA contents in the diet on the mechanical allodynia and neurogenic inflammation that result from CCI-IoN.

## Results

### Weight gain and general behavior

No obvious change could be detected in spontaneous behavior in animals fed different diets, and after CCI or sham surgery. Food intake was also apparently unaffected by diet or surgery, and this was attested by the absence of significant differences in weight gain from six weeks of age to the day of perfusion between the rats fed regular rat chow (RD) (from 218.3 ± 52.0 g to 362.4 ± 27.1 g) and those fed modified diet (MD) (from 198.5 ± 21.9 g to 363.4 ± 19.0 g). Likewise, there were no weight differences at perfusion between the unoperated, naive group (363.4 ± 19.0 g), and the sham (356.4 ± 30.4 g) and CCI groups (340.2 ± 13.5 g).

### Behavioral tests

**Figure 1:**
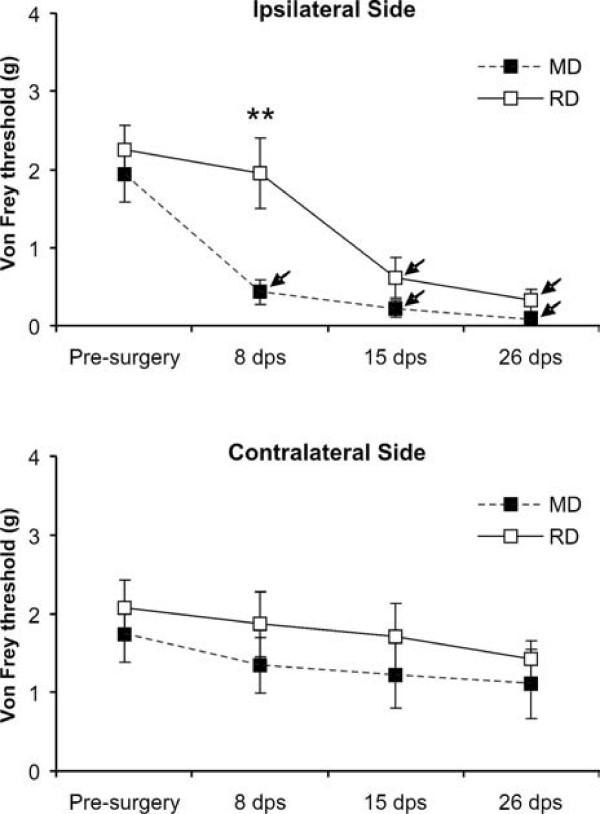
**Diet modifies differently mechanical response thresholds**. Nociceptive responses to Von Frey hairs applied to the vibrissal pads in rats fed RD or MD at pre-surgery time point and at three time intervals after a CCI-IoN performed on the left side. Data represent the mean ± SEM of n = 15 (Pre-surgery and 8 dps) and n = 9 (15 and 26 dps) animals. The unilateral constriction evoked in both diet groups a highly significant decrease in response threshold that evolved with time in the ipsilateral (p < 0.001) but not the contralateral side. Differences between diet groups were highly significant ipsilaterally to the surgery at 8 dps (* p = 0.005), and showed statistical tendency at 15 dps (p = 0.059). Side differences were significant (arrows) at all postsurgery testing times (8 dps, p = 0.008; 15 dps, p = 0.01; 26 dps, p = 0.005) in the MD group, but only at 15 dps (p = 0.04) and 26 dps (p = 0.001) in the RD group.

The baseline response threshold to mechanical stimuli was similar in both diet groups (2.25 ± 0.32 g in RD vs. 1.94 ± 0.35 g in MD, expressed as bending force needed to elicit 50% of positive responses), and exhibited a highly significant decrease after CCI on the ipsilateral (Kruskal-Wallis, p < 0.001) but not the contralateral side in both. However, this decline in threshold appeared earlier and was more pronounced in the MD group, which differed very significantly from the RD group at 8 days postsurgery (0.43 ± 0.16 g vs. 1.96 ± 0.45 g; p = 0.005). At 15 days the difference showed statistical tendency (0.22 ± 0.11 g vs. 0.61 ± 0.26 g; p = 0.059), and by 26 days after surgery a small difference was still observed (0.10 ± 0.04 g vs. 0.32 ± 0.15 g), which did not reach significance. Side comparisons for each force tested also revealed a different time course of mechanical allodynia for RD and MD groups, showing an earlier onset of higher responsiveness in rats fed MD (at day 8 postsurgery), but the same pattern of side differences in MD and RD 26 days after surgery (Fig. 1). In the right side, contralateral to the CCI, the response threshold after surgery was somewhat lower in rats fed MD compared to those fed RD, although this difference did not reach statistical significance.

### Effects of diet on plasma extravasation in naive animals

**Figure 2:**
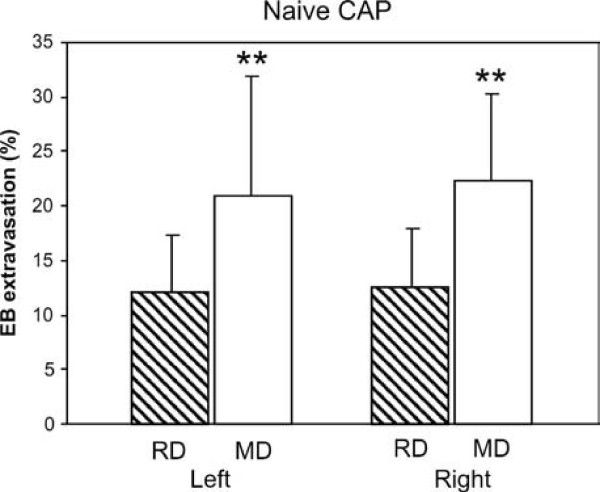
**CAP-induced plasma extravasation is larger in naive rats fed MD**. Extravasation nearly doubled on both sides of the snout in animals maintained on MD (n = 14) compared to those on RD (n = 14; ** p < 0.01). Data represent means ± SD of normalized EB absorbance values (see Methods).

CAP-induced plasma extravasation in unoperated animals kept on MD (n = 14) was almost twice as large as that in the RD group (n = 14; Fig. 2). Pooling both sides for each group, extravasation values were 12.38 ± 5.13 for RD, and 21.57 ± 9.46 for MD. This 72% difference was very significant even when each side was compared separately (p = 0.012 for the left sides, p < 0.001 for the right). Similar values were obtained when comparisons between diet groups were made for the sham-operated animals (9.8 ± 4.4 for RD, n = 9; 24.9 ± 11.7 for MD, n = 9; p = 0.002).

**Figure 3:**
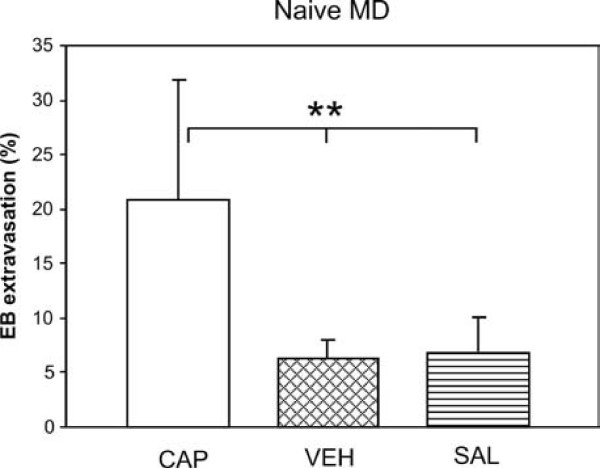
**Extravasation dependence on topical CAP in naive MD groups**. Extravasation measured after application of either VEH (n = 6) or SAL (n = 5) did not reach one-third of the values reached after CAP (n = 14). Only results for the left side are shown (those on the right were essentially the same). Data represent means ± SD. ** p < 0.002.

The effects of topical application of vehicle (VEH) or saline (SAL) instead of CAP on naive rats was examined only in the group fed MD, on the assumption that, in the improbable case that those treatments altered basal extravasation, such effect would be more marked under no "pain-protecting" feeding conditions. Our results (plotted in Fig. 3 for the left side) showed no differences in extravasation (p > 0.60 in all cases) following application of VEH (6.27 ± 1.72) or SAL (6.89 ± 3.09) on either side, while both values differed significantly (p < 0.002) from the extravasation elicited by CAP.

### Effects of CCI-IoN on plasma extravasation

#### Side differences per diet group

**Figure 4:**
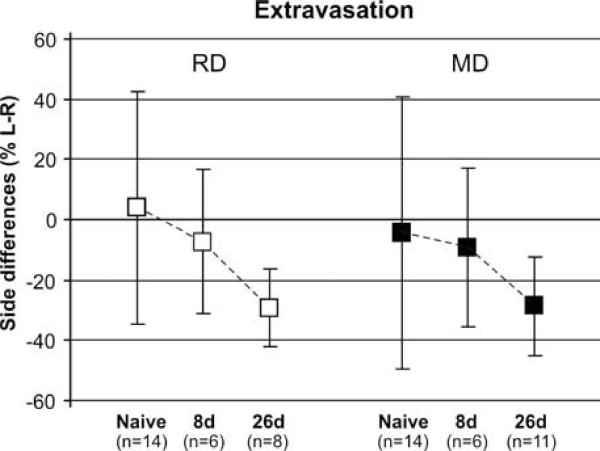
**CAP-induced extravasation tends to decrease with time in the constricted side in both diet groups**. Side comparisons within group showed a progressive relative decrease of extravasation in the constricted side. However, this reduction only reached statistical tendency (p = 0.054) 26 days after surgery in the RD group. Data represent means ± SD.

The average left-right ratio of extravasation for each diet group showed a progressive decrease of extravasation in the constricted side, from a negligible ± 4% in naive groups, to -7% (RD) or -9% (MD) at 8 days after surgery, and -29% (RD) or -28% (MD) at 26 days. However, because of a large interindividual variability, and because of a similar (albeit less pronounced) decreasing trend in the contralateral side, this reduction only reached statistical tendency (p = 0.054) 26 days after surgery in the RD group (Fig. 4).

#### Differences between groups in the CCI (left) side

**Figure 5:**
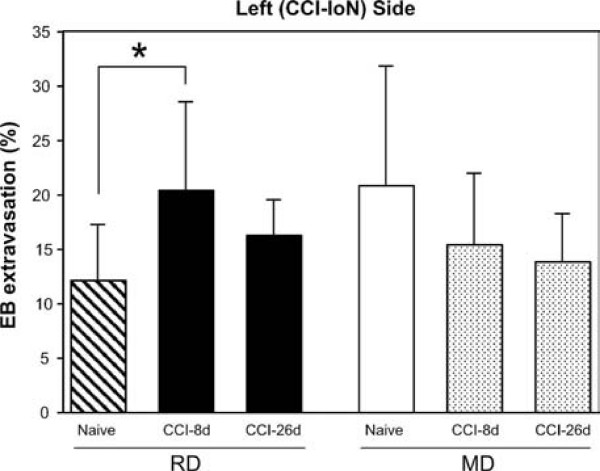
**Extravasation differences between groups on the constricted side**. When compared with results in naive animals fed the same diet, the changes in CAP-induced extravasation generated by the CCI had opposite directions, depending on the diet. In animals fed RD, the values of plasma extravasation increased significantly (p = 0.043) after surgery. This increase was most marked at 8 dps (* p = 0.017), and more moderate at 26 dps (showing statistical tendency, p = 0.052). In contrast, in animals fed MD the extravasation decreased with respect to naive animals, and this decrease showed statistical tendency at 26 dps (p = 0.056). No significant differences were found in lesioned rats on the side of the CCI between RD and MD groups at any of the postsurgical times tested. Data represent means ± SD; number of cases as in Fig. 4.

In the RD group, CAP-induced plasma extravasation increased significantly after surgery with respect to both naive (Kruskal-Wallis, p = 0.043) and sham-operated groups (ANOVA, p < 0.001). Compared to the naive animals (Fig. 5), this increase reached a significant 68% at 8 days after surgery (* p = 0.017), but displayed a more moderate 35% at 26 dps (showing statistical tendency, p = 0.052). In contrast, in animals fed MD the extravasation decreased with respect to naive animals. This decrease was a non-significant 26% at 8 dps, but reached 34% at 26 dps (p = 0.056). Compared to the sham-operated rats, the extravasation increases observed in the RD group became highly significant at 8 (p < 0.002) and 26 dps (p < 0.001), whereas the moderate decreases detected in the MD group remained below the significance level.

These findings indicate that the CCI elicited changes on CAP-induced extravasation in opposite directions, which depended on the different basal levels that were characteristic of each diet. Moreover, no significant differences were found on the side of the CCI between RD and MD groups at any postsurgical time, suggesting that, at least by 8 days after CCI, the nerve injury has already imposed a certain degree of CAP-induced extravasation, regardless the initial, diet-dependent conditions.

#### Differences between groups in the contralateral (right) side

**Figure 6:**
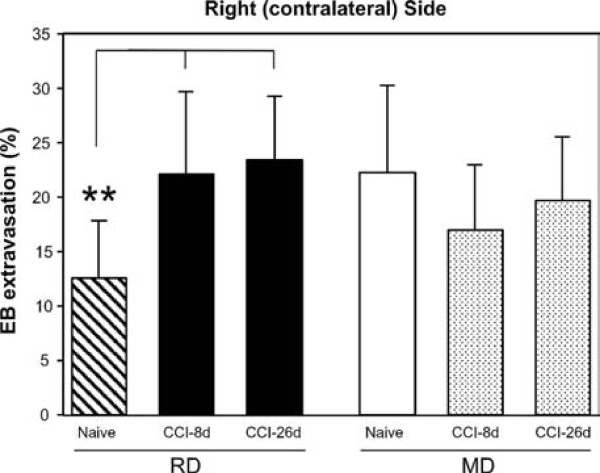
**Extravasation differences between groups on the side contralateral to the constriction**. The CAP-induced extravasation on the right vibrissal pad in animals fed RD increased very significantly (** p < 0.006) at 8 and 26 dps following a CCI of the left IoN. In contrast, no differences between naive and CCI-IoN groups were noticed in animals fed MD, which in all cases displayed high levels of extravasation on the right side. No significant differences were found on the contralateral side of the CCI between RD and MD groups at any of the postsurgical times tested. Data represent means ± SD; number of cases as in Fig. 4.

Following a unilateral CCI-IoN, the notable diet-dependent difference in CAP-induced extravasation found in naive rats also disappeared in the opposite side of the constriction. This was due to a large increase in extravasation on the right vibrissal pad in animals fed RD at 8 (+75%, p < 0.006) and 26 dps (+85%, p < 0.001), while only minor (-23% and -12%, respectively) and non-significant reductions from the basal high levels of extravasation found in naive rats were observed in the right side of animals fed MD (Fig. 6). This resulted in no significant differences between RD and MD groups at any of the postsurgical times tested. Similar results were obtained when comparisons were made with the sham-operated animals, except for somewhat larger differences with the operated groups, which reached high significance in the RD group, and statistical tendency (0.05 < p < 0.08) in the MD group.

## Discussion

In this study we aimed at investigating the effects of the CCI-IoN on mechanical allodynia and neurogenic inflammation in rats fed a standard diet, or a modified diet with a very low content of PUFAs. The main findings of this study are: 1, the mechanical allodynia in the trigeminal territory following CCI-IoN is more pronounced, and develops 1–2 weeks earlier when rats were deprived of PUFAs in the diet; 2, CAP-induced plasma extravasation in naive rats fed MD is nearly twice as large as in those fed RD; 3, eight days after CCI-IoN the extravasation in the operated side reaches similar levels in both diet groups; 4, the CCI also causes a contralateral rise of extravasation, but only in the RD group, bringing its levels to the same range of those in the MD group; and 5, consequently, the net effect of CCI-IoN on extravasation is a moderate reduction in the MD group, but a significant increase in the RD group, compared with basal levels (from naive and/or sham cases).

### CCI-IoN and neurogenic inflammation

Nerve injury underlying different neuropathic conditions is known to reduce the flare response to topical application of substance P, histamine or capsaicin [28,29]. A related effect, consisting of reduction of vasodilatation and decreased plasma extravasation, has been reported in widely used models of neuropathy in the sciatic territory in rats, such as the CCI of the sciatic nerve [9,11] or the spinal nerve ligation [30]. The axon reflex-dependent vasodilatation is normally mediated by antidromic activation of a subpopulation of Aδ and C fibers [8,10], but Aβ fibers activated from injured or irritated target tissues may also play a role, by centrally sensitizing C nociceptors [31]. Plasma extravasation, however, depends not on Aδ fibers [8], but on C fibers, because it is elicited by specific antidromic stimulation of polymodal C fibers [32,33], and is prevented by specifically blocking axoplasmic transport in C fibers with colchicine [34].

We have shown that topical application of CAP to the vibrissal pad also elicits neurogenic extravasation, which is reduced ipsilaterally to a CCI-IoN. However, under standard dietetic conditions, the actual levels of plasma extravasation are increased *bilaterally *after performing a unilateral constriction. This results in a net extravasation *increase *in the constricted side, when compared with the same side in naive or sham groups. To our knowledge, similar intergroup comparisons are mostly lacking concerning extravasation after chronic constriction or partial nerve injury, not only in trigeminal fields, but also in the more commonly studied sciatic or saphenous territories. However, Yonehara and Yoshimura [11] showed a significant net reduction in EB released by CAP application or nerve stimulation into the perfusate collected through a subcutaneous cannula, 7 days after sciatic CCI. This difference with our results may be due not only to a local damage created by the inserted cannula in that study, but also to a different degree of nerve damage between the sciatic and the trigeminal CCI models. Yonehara and Yoshimura [11] used Bennett and Xie's [7] four-ligature model of sciatic CCI, which is reported to determine a nearly complete loss of myelinated fibers and a substantial loss of unmyelinated axons, two weeks after ligation [35]. A similar quantitative analysis is not yet available for the CCI-IoN model, which used two loose ligatures [5], nor for our variant, which used a single ligature. Qualitative observations by Anderson et al. [36] after a single ligature, however, suggested a relatively moderate fiber loss three weeks after CCI-IoN. In contrast, we have found that four relatively tight ligatures applied to the IoN resulted in a substantial fiber loss and a marked net reduction of EB extravasation, but also -as previously reported by others [37] – a less pronounced and less consistent mechanical allodynia (unpublished findings).

### Contralateral effects of nerve injury on extravasation

The appearance of contralateral effects after a unilateral nerve injury has been known for a number of years. They consist of a diversity of phenomena, from altered gene expression to a range of functional and anatomical changes in homotopical contralateral peripheral nerves, sensory ganglia or motoneurons [38]. Acute contralateral neurogenic responses, such as tissue edema or plasma extravasation into the synovial space, have been described following the induction of unilateral inflammation [39-41]. More recently, Kelly et al. [42] showed that five days after unilateral induction of inflammation in the rat knee joint, EB extravasation in the contralateral knee joint was increased, and this increase was accompanied by an elevated rate of spontaneous activity in CAP-sensitive fibers of the saphenous nerve (just above the incorporation of the medial articular nerve). These observations are consistent with our finding of an increased EB extravasation in the vibrissal pad contralateral to the CCI-IoN. Although a conclusive explanation for the contralateral effects is yet to be obtained, it has been shown that a pharmacological blockade of C and Aδ nociceptive fibers and/or sympathetic fibers in the sciatic nerve blocks the sustained peripheral vasodilatation elicited by a contralateral sciatic CCI [43], supporting the early claim that both neural components participate in what was called reflex neurogenic inflammation [39]. The role of the sympathetic system for explaining the increase in peripheral extravasation after partial limb nerve lesion is debated [30,44], but it has been shown that SNL induces sympathetic fibers sprouting not only in the ipsilateral but also the contralateral DRG [45-47]. Less likely, however, would be a similar role for the sympathetic innervation in the trigeminal territory after unilateral CCI-IoN, given the absence of sympathetic fiber sprouting in the trigeminal ganglion after nerve injury (IoN or inferior alveolar nerve constriction: [24]). Moreover, the fraction of sympathetic fibers composing the trigeminal nerve is 2.5 times lower than in limb nerves [48].

### Effects of dietary PUFAs on nociceptive behavior and neurogenic inflammation

The use of corn or soy as sources of dietary fat has been associated to decreased expression of mechanical allodynia following partial sciatic nerve ligation (PSL, [16-18]). Both types of fat contain similarly high levels of linoleic acid (58%), a short chain (18 carbons) PUFA of the ω-6 family with 2 double bonds, but different levels of α-linolenic acid (0.7% in corn, 6.8% in soy), a short chain PUFA of the ω-3 family with 3 double bonds [19,49]. All the above mentioned studies tested the rats for the presence of allodynia for up to 10–14 days after PSL. After CCI-IoN we confirmed that the long-term absence of PUFAs from the diet elicited a decrease in mechanoresponsive thresholds, and an overt mechanical allodynia 8 and 15 days postsurgery, as judged from comparisons with responses from the contralateral, undamaged side. At 15 days, rats fed RD also started to display allodynia, but with response thresholds still higher than rats fed MD. By 26 days postconstriction, however, the allodynia reached the same level irrespective of the diet used, suggesting that the "protective" effect of the PUFAs-rich diet would be temporary. In the absence of a more prolonged follow-up, it remains to be investigated whether the total duration of CCI-connected neuropathic symptoms is affected by dietary lipid content.

The removal of PUFAs from the diet also had a hitherto unreported effect on neurogenic inflammation, consisting of, 1, an increase of CAP-induced plasma extravasation in the vibrissal pads of uninjured animals, and 2, an ipsilateral reduction following CCI-IoN, while maintaining high levels of extravasation in the contralateral side. Although the mechanisms of this effect remain elusive, the relationships existing between dietary lipids and inflammation may shed some light on them. Dietary fatty acids, which have been used to treat different inflammatory conditions [20,22], have a complex influence on inflammatory responses, which is mainly exerted by modifying the production of inflammation-related eicosanoids and cytokines. The simplest types of unsaturated fatty acids of the ω-6 and ω-3 families cannot be synthesized in mammals, but once ingested, are desaturated further and elongated, giving rise to a series of long chain PUFAs, with different, and in some aspects opposite effects on inflammatory processes [19,50]. While the ω-6 PUFAs boost the production of potent proinflammatory eicosanoids (such as prostaglandins and leukotrienes) and cytokines (such as interleukins 1 and 6, and TNFα), ω-3 PUFAs antagonize inflammatory responses by altering the eicosanoid production through metabolic competition with the ω-6 PUFAs, by indirectly blocking the expression of proinflammatory genes such as NF-κB, or by generating antiinflammatory mediators, such as the recently described resolvins, docosatrienes and neuroprotectins [50,51].

Dietary ω-3 PUFAs also alleviate chronic pain by mechanisms other than their antiinflammatory effects, including inhibition of neuronal protein kinases and blocking of voltage-gated calcium channels involved in both inflammatory and NP processing, general reduction of the sympathetic tone, and, perhaps, improvement of pain-associated affective disorders and other psychiatric conditions through largely unexplained mechanisms [20,52-54]. Moreover, the absence of dietary α-linolenic acid may enhance the formation of lysophosphatidic acid [55], which is considered a critical mediator in the development of NP, through direct actions on the primary sensory neurons [56,57], and by inducing demyelination and ephaptic cross-talk between fibers in dorsal roots and peripheral nerves [58].

In contrast, because of their putative proinflammatory effects, the possible effects on pain control of dietary ω-6 PUFAs have been essentially neglected. However, a clear-cut opposite effect of dietary short chain PUFAs on pain is questionable. Firstly, in naive rats it was an appropriate ratio of ω-3:ω-6 fatty acids in diet (around 1:5), rather than the absolute amount of ω-3 intake, which was associated to an elevation of the withdrawal threshold to acute thermal noxious stimuli [13,21]. This is consistent with the fact that, because of the complex metabolic interaction between the ω-families of dietary fatty acids, their final outcome on the tissue fatty acid composition depends more on the ratios between their different classes than on their absolute amounts in the diet [19]. Secondly, a significant positive correlation was recently reported between the total intake of α-linolenic acid (an ω-3 PUFA) and thermal hyperalgesia (but not mechanical allodynia) after a PSL [49]. Moreover, the presence of both ω-3 and ω-6 PUFAs' families in the intake could help reduce the excitability of sodium channels involved in pain [59], particularly in injured nerves [60]. In our study, the lipid content of the olive oil used in MD to replace the fat present in RD had just one-eighth of linoleic acid (6.3% vs. 50%), and practically lacked α-linolenic acid (traces vs. 4.5%; [61]), making olive oil a well-suited placebo in studies of the antiinflammatory effect of PUFAs [62].

## Conclusion

In summary, our findings demonstrate that dietary fat rich in PUFAs reduces CAP-induced neurogenic plasma extravasation in the trigeminal territory, and that a sustained removal of PUFAs intake significantly alters the mechanical allodynia and the plasma extravasation that result from a unilateral CCI-IoN. In addition, the observation of contralateral effects of CCI-IoN, together with the existence of natural asymmetries in some systems, warrant caution against uncritically using the contralateral side as control [63,64].

## Methods

### Experimental Animals

Adult male Sprague-Dawley rats (n = 108) from our own colony, originating from Harlan (Harlan Iberica, Barcelona), were used. They were divided into 14 groups, combining the various treatment protocols: Unilateral (left) CCI-IoN, sham operation, or no surgery (naive); bilateral application of CAP cream (1.6%), VEH (base cream) or SAL to the vibrissal pad; and maintenance on RD or MD. Animals' weight was recorded at six weeks of age, at the surgery day and at the perfusion day.

Rats were housed under standard colony conditions (4 rats per cage). Food and water were supplied ad libitum, and the animals were kept under a reversed 12:12 h dark/light cycle. All experiments were carried out in accordance with the Guidelines on Ethical Standards for Investigation of Experimental Pain in Animals [65] and the European Community's Council Directive 86/609/EEC, and the study was approved by the Ethical Committee of our institution.

### Surgery

Animals were i.m. anesthetized with a mixture of ketamine 55 mg/Kg, xylazine 15 mg/Kg, and atropine 0.2 mg/Kg. The left IoN was exposed as described before [66,67]. One single ligature was tied loosely around the distal part of the nerve using a polypropylene monofilament suture (Surgipro 6.0). The mild degree of constriction apparently did not block the circulation through the superficial epineural vasculature of the IoN, as proposed by Bennett and Xie [7] for the sciatic CCI, although the fan-like geometry of the multi-fascicled IoN undoubtedly resulted in different degrees of constriction on different fascicles. Special care was taken to avoid an excessive compression of the nerve, which hampered the development of allodynia and resulted in more severe fiber loss (results not shown). The wound was closed using silk sutures. In sham-operated rats the IoN was exposed on the left side using the same procedure but the nerve was left untouched.

### Diet

Two types of diet were used. The RD was the normal rat chow provided by the animal holding facility (SAFE-A04, Epinay-sur-Orge, France), containing 3.1% lipids derived from barley, corn, soy, fish by-products and wheat. More than half of the lipid content (54.5%) was the PUFAs linoleic acid (50%) and α-linolenic acid (4.5%). The MD (Nutreco España, Toledo, Spain) was prepared *ad hoc *for this study, and consisted of pellets of similar texture and composition, except for the source of lipids (6%), which were provided exclusively by olive oil, which contains a much lower proportion of PUFAs (6.3% linoleic acid, and traces of linolenic acid). Protein content in both diets derived mostly from barley, wheat and soy, and reached roughly the same levels (16%). Six week-old rats were exposed to either RD or MD for two additional weeks before the surgeries. Each group was maintained on the corresponding diet until the end of the experiment.

### Behavioral Tests

Before every stimulation session, rats were habituated for one hour daily to the testing room environment during 2–3 days. The animals were placed in clear acrylic cages (30 × 30 × 20 cm) in order to reduce their field of exploration. When the rat was still, with the four paws placed on the ground, it was gently handled to ensure that it became familiar with the experimenter and the approximation of the test filaments, in order to minimize stress.

Tactile sensitivity in the vibrissal area was measured with a set of eight calibrated von Frey monofilaments (North Coast Medical, Inc. Morgan Hill, CA) ranging from 0.02 g to 4.0 g. Each filament was applied to the bending point five times at 15–30 s intervals, on five different points of each vibrissal pad. The stimulus intensity was presented in a sequential ascending order. The lowest force that evoked 50% of withdrawal responses was considered the threshold. Responses to mechanical stimuli were explored bilaterally before the CCI surgery, and 8, 15 and 26 days after surgery.

### Determination of plasma extravasation

Eight and twenty-six days after CCI-IoN, and 1–2 days following the last behavioral test, rats were anesthetized with pentobarbital (Dolethal, 50 mg/Kg, i.p.), had their snouts shaved, and then they received an i.v. injection of EB (Sigma; 50 mg/Kg of a 50 mg/ml solution in 0.9% saline) in the tail vein. Immediately after injection, the appropriate topical treatment (CAP, VEH, SAL) was applied bilaterally on the whisker pad. After 10 minutes the animals were perfused through the ascending aorta with saline (0.9% NaCl), and the trigeminal pads were quickly removed and transferred to an oven at 56°C for 2 days. Formamide (Sigma) was used to extract the EB out of the tissue [34], and local extravasation was estimated from the EB concentration measured with a spectrophotometer (Molecular Devices) at a wavelength of 620 nm. Because of the inherent intertrial variability, it was necessary to create a standard curve for each experimental session, against which the experimental results could be normalized. The absorbance values over a range of EB-formamide concentrations were adjusted to a sigmoidal curve through non-linear regression, and the concentration of EB corresponding to saturation was used as 100% reference for all experimental values obtained within the same session. The group means and dispersion values were then represented as percentages of saturation. Moreover, various animals from different groups were coded and processed in every measuring session.

### Animal coding and statistics

After CCI surgery animals were coded by subcutaneous electronic tags, so that all behavioral tests and EB procedures were carried out with the experimenter blinded to the type of diet consumed and treatment received by the rats. For all statistical analysis Statgraphics Plus 4.0 were used. Side comparisons within the same group were made with a two-tailed t-test. For intergroup comparisons with factors treatment (CAP, VEH or SAL) and manipulation (CCI, sham or naive) a two-way analysis of variance (ANOVA) was applied when parametric conditions were met, or the non-parametric Kruskal-Wallis test otherwise. If multiple comparisons yielded significance, two-by-two comparisons were made with a t-test or Wilcoxon, as appropriate. The significance level was set at p < 0.05.

## Abbreviations

CAP: capsaicin; CCI: Chronic constriction injury; dps: days post-surgery; DRG: dorsal root ganglia; EB: Evans Blue; IoN: infraorbital nerve; MD: modified diet; NP: neuropathic pain; PSL: partial sciatic nerve ligation; PUFA: polyunsaturated fatty acids; RD: regular diet; SAL: saline; VEH: vehicle.

## Competing interests

The authors declare that they have no competing interests.

## Authors' contributions

YBM carried out the experiments and surgery, performed the statistical analyses, and contributed to the writing of the paper. CA played a major role in designing the study, preparing the modified diet, composing the Figures and writing the paper. Both authors discussed, read and approved the final manuscript.

## References

[B1] Macfarlane TV, Blinkhorn AS, Davies RM, Ryan P, Worthington HV, Macfarlane GJ (2002). Orofacial pain: just another chronic pain? Results from a population-based survey. Pain.

[B2] Campbell JN, Meyer RA (2006). Mechanisms of neuropathic pain. Neuron.

[B3] Zhuo M (2007). Neuronal mechanism for neuropathic pain. Mol Pain.

[B4] Koltzenburg M, Hunt SP, Koltzenburg M (2005). Neuropathic pain. Neurobiology of Pain.

[B5] Vos BP, Strassman AM, Maciewicz RJ (1994). Behavioral evidence of trigeminal neuropathic pain following chronic constriction injury to the rat's infraorbital nerve. J Neurosci.

[B6] Benoist JM, Gautron M, Guilbaud G (1999). Experimental model of trigeminal pain in the rat by constriction of one infraorbital nerve: changes in neuronal activities in the somatosensory cortices corresponding to the infraorbital nerve. Exp Brain Res.

[B7] Bennett GJ, Xie YK (1988). A peripheral mononeuropathy in rat that produces disorders of pain sensation like those seen in man. Pain.

[B8] Jänig W, Lisney SJ (1989). Small diameter myelinated afferents produce vasodilatation but not plasma extravasation in rat skin. J Physiol.

[B9] Basile S, Khalil Z, Helme RD (1993). Skin vascular reactivity to the neuropeptide substance P in rats with peripheral mononeuropathy. Pain.

[B10] Gee MD, Lynn B, Cotsell B (1997). The relationship between cutaneous C fibre type and antidromic vasodilatation in the rabbit and the rat. J Physiol.

[B11] Yonehara N, Yoshimura M (2001). Influence of painful chronic neuropathy on neurogenic inflammation. Pain.

[B12] Frye CA, Bock BC, Kanarek RB (1992). Hormonal milieu affects tailflick latency in female rats and may be attenuated by access to sucrose. Physiol Behav.

[B13] Yehuda S, Carasso RL (1993). Modulation of learning, pain thresholds, and thermoregulation in the rat by preparations of free purified alpha-linolenic and linoleic acids: determination of the optimal omega 3-to-omega 6 ratio. Proc Natl Acad Sci USA.

[B14] Zhang T, Reid K, Acuff CG, Jin CB, Rockhold RW (1994). Cardiovascular and analgesic effects of a highly palatable diet in spontaneously hypertensive and Wistar-Kyoto rats. Pharmacol Biochem Behav.

[B15] Ren K, Blass EM, Zhou Q, Dubner R (1997). Suckling and sucrose ingestion suppress persistent hyperalgesia and spinal Fos expression after forepaw inflammation in infant rats. Proc Natl Acad Sci USA.

[B16] Shir Y, Sheth R, Campbell JN, Raja SN, Seltzer Z (2001). Soy-containing diet suppresses chronic neuropathic sensory disorders in rats. Anesth Analg.

[B17] Shir Y, Ratner A, Raja SN, Campbell JN, Seltzer Z (1998). Neuropathic pain following partial nerve injury in rats is suppressed by dietary soy. Neurosci Lett.

[B18] Pérez J, Ware MA, Chevalier S, Gougeon R, Bennett GJ, Shir Y (2004). Dietary fat and protein interact in suppressing neuropathic pain-related disorders following a partial sciatic ligation injury in rats. Pain.

[B19] Kelley DS (2001). Modulation of human immune and inflammatory responses by dietary fatty acids. Nutrition.

[B20] Shapiro H (2003). Could n-3 polyunsaturated fatty acids reduce pathological pain by direct actions on the nervous system?. Prostaglandins Leukot Essent Fatty Acids.

[B21] Yehuda S, Leprohon-Greenwood CE, Dixon LM, Coscina DV (1986). Effects of dietary fat on pain threshold, thermoregulation and motor activity in rats. Pharmacol Biochem Behav.

[B22] Goldberg RJ, Katz J (2007). A meta-analysis of the analgesic effects of omega-3 polyunsaturated fatty acid supplementation for inflammatory joint pain. Pain.

[B23] Tal M, Devor M (1992). Ectopic discharge in injured nerves: comparison of trigeminal and somatic afferents. Brain Res.

[B24] Bongenhielm U, Boissonade FM, Westermark A, Robinson PP, Fried K (1999). Sympathetic nerve sprouting fails to occur in the trigeminal ganglion after peripheral nerve injury in the rat. Pain.

[B25] Fried K, Bongenhielm U, Boissonade FM, Robinson PP (2001). Nerve injury-induced pain in the trigeminal system. Neuroscientist.

[B26] Ambalavanar R, Moritani M, Dessem D (2005). Trigeminal P2X3 receptor expression differs from dorsal root ganglion and is modulated by deep tissue inflammation. Pain.

[B27] Davies SL, Loescher AR, Clayton NM, Bountra C, Robinson PP, Boissonade FM (2006). Changes in sodium channel expression following trigeminal nerve injury. Exp Neurol.

[B28] Aronin N, Leeman SE, Clements RS (1987). Diminished flare response in neuropathic diabetic patients. Comparison of effects of substance P, histamine, and capsaicin. Diabetes.

[B29] Morris GC, Gibson SJ, Helme RD (1995). Capsaicin-induced flare and vasodilatation in patients with post-herpetic neuralgia. Pain.

[B30] Kauppila T, Kontinen VK, Wei H, Jyväsjärvi E, Pertovaara A (2002). Cutaneous vascular responses evoked by noxious stimulation in rats with the spinal nerve ligation-induced model of neuropathy. Brain Res Bull.

[B31] Garcia-Nicas E, Laird JM, Cervero F (2001). Vasodilatation in hyperalgesic rat skin evoked by stimulation of afferent A beta-fibers: further evidence for a role of dorsal root reflexes in allodynia. Pain.

[B32] Kenins P (1981). Identification of the unmyelinated sensory nerves which evoke plasma extravasation in response to antidromic stimulation. Neurosci Lett.

[B33] Wiesenfeld-Hallin Z (1988). Partially overlapping territories of nerves to hindlimb foot skin demonstrated by plasma extravasation to antidromic C-fiber stimulation in the rat. Neurosci Lett.

[B34] Kingery WS, Guo TZ, Poree LR, Maze M (1998). Colchicine treatment of the sciatic nerve reduces neurogenic extravasation, but does not affect nociceptive thresholds or collateral sprouting in neuropathic or normal rats. Pain.

[B35] Basbaum AI, Gautron M, Jazat F, Mayes M, Guilbaud G (1991). The spectrum of fiber loss in a model of neuropathic pain in the rat: an electron microscopic study. Pain.

[B36] Anderson LC, Vakoula A, Veinote R (2003). Inflammatory hypersensitivity in a rat model of trigeminal neuropathic pain. Arch Oral Biol.

[B37] Anderson LC, Rao RD (2001). Interleukin-6 and nerve growth factor levels in peripheral nerve and brainstem after trigeminal nerve injury in the rat. Arch Oral Biol.

[B38] Koltzenburg M, Wall PD, McMahon SB (1999). Does the right side know what the left is doing?. Trends Neurosci.

[B39] Levine JD, Dardick SJ, Basbaum AI, Scipio E (1985). Reflex neurogenic inflammation. I. Contribution of the peripheral nervous system to spatially remote inflammatory responses that follow injury. J Neurosci.

[B40] Kidd BL, Cruwys SC, Garrett NE, Mapp PI, Jolliffe VA, Blake DR (1995). Neurogenic influences on contralateral responses during experimental rat monoarthritis. Brain Res.

[B41] Bileviciute I, Stenfors C, Theodorsson E, Lundeberg T (1998). Unilateral injection of calcitonin gene-related peptide (CGRP) induces bilateral oedema formation and release of CGRP-like immunoreactivity in the rat hindpaw. Br J Pharmacol.

[B42] Kelly S, Dunham JP, Donaldson LF (2007). Sensory nerves have altered function contralateral to a monoarthritis and may contribute to the symmetrical spread of inflammation. Eur J Neurosci.

[B43] Kurvers HA, Tangelder GJ, De Mey JG (1996). Skin blood flow disturbances in the contralateral limb in a peripheral mononeuropathy in the rat. Neuroscience.

[B44] Miao FJ, Levine JD (1999). Neural and endocrine mechanisms mediating noxious stimulus-induced inhibition of bradykinin plasma extravasation in the rat. J Pharmacol Exp Ther.

[B45] McLachlan EM, Jänig W, Devor M, Michaelis M (1993). Peripheral nerve injury triggers noradrenergic sprouting within dorsal root ganglia. Nature.

[B46] Chung K, Kim HJ, Na HS, Park MJ, Chung JM (1993). Abnormalities of sympathetic innervation in the area of an injured peripheral nerve in a rat model of neuropathic pain. Neurosci Lett.

[B47] Deng YS, Zhong JH, Zhou XF (2000). BDNF is involved in sympathetic sprouting in the dorsal root ganglia following peripheral nerve injury in rats. Neurotox Res.

[B48] Hoffmann KD, Matthews MA (1990). Comparison of sympathetic neurons in orofacial and upper extremity nerves: implications for causalgia. J Oral Maxilofac Surg.

[B49] Pérez J, Ware MA, Chevalier S, Gougeon R, Shir Y (2005). Dietary omega-3 fatty acids may be associated with increased neuropathic pain in nerve-injured rats. Anesth Analg.

[B50] Calder PC (2005). Polyunsaturated fatty acids and inflammation. Biochem Soc Trans.

[B51] Serhan CN (2005). Novel eicosanoid and docosanoid mediators: resolvins, docosatrienes, and neuroprotectins. Curr Opin Clin Nutr Metab Care.

[B52] Malmberg AB (2000). Protein kinase subtypes involved in injury-induced nociception. Prog Brain Res.

[B53] Sawynok J, Esser MJ, Reid AR (2001). Antidepressants as analgesics: an overview of central and peripheral mechanisms of action. J Psychiatry Neurosci.

[B54] Owen C, Rees AM, Parker G (2008). The role of fatty acids in the development and treatment of mood disorders. Curr Opin Psychiatry.

[B55] Miyazawa D, Ikemoto A, Fujii Y, Okuyama H (2003). Dietary alpha-linolenic acid suppresses the formation of lysophosphatidic acid, a lipid mediator, in rat platelets compared with linoleic acid. Life Sci.

[B56] Inoue M, Rashid MH, Fujita R, Contos JJ, Chun J, Ueda H (2004). Initiation of neuropathic pain requires lysophosphatidic acid receptor signaling. Nat Med.

[B57] Lee WS, Hong MP, Kim TH (2005). Effects of lysophosphatidic acid on sodium currents in rat dorsal root ganglion neurons. Brain Res.

[B58] Ueda H (2008). Peripheral mechanisms of neuropathic pain – involvement of lysophosphatidic acid receptor-mediated demyelination. Mol Pain.

[B59] Baker MD, Wood JN (2001). Involvement of Na+ channels in pain pathways. Trends Pharmacol Sci.

[B60] Hong MP, Kim HI, Shin YK, Lee CS, Park M, Song JH (2004). Effects of free fatty acids on sodium currents in rat dorsal root ganglion neurons. Brain Res.

[B61] (2003). Tablas FEDNA de composición y valor nutritivo de alimentos para la fabricación de piensos compuestos.

[B62] Calder PC (2006). n-3 polyunsaturated fatty acids, inflammation, and inflammatory diseases. Am J Clin Nutr.

[B63] Lagares A, Avendaño C (2000). Lateral asymmetries in the trigeminal ganglion of the male rat. Brain Res.

[B64] Scott C, Perry MJ, Raven PE, Massey EJ, Lisney SJ (2000). Capsaicin-sensitive afferents are involved in signalling transneuronal effects between cutaneous sensory nerves. Neuroscience.

[B65] Zimmermann M (1983). Ethical guidelines for investigations of experimental pain in conscious animals. Pain.

[B66] Gregg JM, Dixon AD (1973). Somatotopic organization of the trigeminal ganglion in the rat. Arch Oral Biol.

[B67] Jacquin MF, Zeigler HP (1983). Trigeminal orosensation and ingestive behavior in the rat. Behav Neurosci.

